# Acute Activity Urges Predict Lower Early Weight Gain During Inpatient Treatment for Anorexia Nervosa

**DOI:** 10.1002/eat.24305

**Published:** 2024-10-18

**Authors:** Georg Halbeisen, Lina Amin, Karsten Braks, Thomas J. Huber, Georgios Paslakis

**Affiliations:** ^1^ University Clinic for Psychosomatic Medicine and Psychotherapy, Medical Faculty, Campus East‐Westphalia Ruhr‐University Bochum Luebbecke Germany; ^2^ Centre for Eating Disorders, Klinik Am Korso Bad Oeynhausen Germany

**Keywords:** anorexia nervosa, inpatient treatment, physical activity, psychotherapy, restlessness, urge for movement

## Abstract

Early weight gain is a primary goal in the treatment of anorexia nervosa (AN) and associated with more favorable discharge weights and clinical outcomes. Activity urges, that is, a motivational state to engage in activity, have been suspected to delay early weight gain, but their prognostic role remains barely explored. Here, we investigated whether acute (state‐like) activity urges at treatment onset would predict within‐person weight gain in patients with AN during the initial 2 weeks of inpatient treatment. Adults with AN from an inpatient unit (*N* = 53) completed an activity urges measure at treatment onset, and weight changes were monitored for the duration of their treatment. Regression analyses, controlling for admission body mass index and other patient variables (i.e., patient age and AN subtype), found that higher state activity urges were associated with lower initial weight gain. Mediation analyses showed that differences in early weight changes further linked higher activity urges at admission to lower discharge weights. An activity urge cutoff value of 2.76 for distinguishing between cases with optimal and suboptimal initial weight gain is proposed. We discuss potential mechanisms of the link between activity urges and early weight gain and the implications of activity urges as a prognostic factor for improving weight restoration during AN treatment.

**Summary**

- Anorexia nervosa is a severe mental disorder with high mortality rates.
- There is a continuing need to identify factors associated with weight restoration in patients with anorexia nervosa.
- This study suggests the subjective urge to engage in physical activities to be of prognostic significance and discusses implications for treatment.

## 1 Introduction

Anorexia nervosa (AN) is a severe mental disorder characterized by significant weight loss or failure to gain weight appropriately for age due to restrictions of energy intake, an intense fear of weight gain, and a disturbed body image (American Psychiatric Association 2013). Its variants include a binge‐eating/purging subtype characterized by binge‐eating or purging episodes (e.g., self‐induced vomiting), a restricting subtype without binge‐eating and purging, as well as “atypical” forms that do not fulfill the full range of diagnostic criteria (e.g., patients who are not underweight, or who do not fear weight gain). AN is comparatively rare, with a global lifetime prevalence of 1.4% in young women and girls (vs. 0.2% in boys and men; Galmiche et al. 2019). However, AN is one of the most lethal mental disorders, in part due to severe underweight (Cuntz, Quadflieg, and Voderholzer 2023). Patients with AN have a 5.9 times higher all‐cause mortality risk compared to the general population (Chesney, Goodwin, and Fazel 2014). Weight restoration is thus a primary goal in AN treatment, especially during early stages of recovery (Herpertz‐Dahlmann et al. 2015). Indeed, early weight gain, within the first few weeks of treatment, has been linked to higher discharge weight and positive clinical outcomes across various treatment settings (Austin et al. 2021; Chatelet et al. 2020; Halbeisen et al. 2024; Hartmann, Wirth, and Zeeck 2007; Kolar et al. 2022; Le Grange et al. 2014; Mewes, Tagay, and Senf 2008; Wade et al. 2021; Wales et al. 2016). Identifying and targeting factors beneficial or detrimental to early weight gain could thus be of great therapeutic effect (Nazar et al. 2017).

Evidence‐based guidelines for AN treatment recommend at least 500 g of weekly weight gain for inpatients (and 200 g for outpatients; Hilbert, Hoek, and Schmidt 2017); tightly controlled settings are necessary to achieve weight gain (Zeeck et al. 2018). A suspected reason for delayed weight gain in patients with AN, especially during early treatment, is the urge to engage in physical activity (PA) (Graap, Erim, and Paslakis 2018; Marzola et al. 2013; Paslakis et al. 2017). PA describes any body movement produced by skeletal muscle contractions as part of exercising and nonexercising behaviors (e.g., standing, walking, or fidgeting). Because PA contributes to maintaining low body weight by increasing energy expenditure relative to basal metabolism (Chung et al. 2018; Elbelt et al. 2013; Haas et al. 2018; Jakicic 2009; Westerterp 2013), abstinence from PA is often recommended during the initial treatment stages of AN (Quesnel et al. 2018). Still, up to 80% of patients with AN experience an increased urge to engage in PA irrespective of restricting or binge‐eating/purging subtype and despite fatigue or tiredness (Casper et al. 2020; Gümmer et al. 2015; Keyes et al. 2015). Rizk et al. (2020) suggested that the psychological urge for PA develops gradually, with patients initially engaging in PA to maximize weight loss, which then transitions into a coping strategy to alleviate negative affect and ultimately evolves into an autonomous activity drive characterized by diffuse restlessness and involuntary movement (Casper 2020; Casper et al. 2020). Since activity urges, that is, a motivational state to engage in activity, may promote both exercising and nonexercising behaviors (e.g., fidgeting), they may also be linked to increased energy expenditure and thus lower early weight gain.

Thus far, few studies have explored whether activity urges could be linked to early weight gain in patients with AN. Objective PA levels, measured using actigraphy, predicted weight gain in patients with AN only inconsistently. Using a proprietary software that calculated objective PA levels from a wrist‐worn accelerometer with skin temperature and conductance measures, Kemmer et al. (2020) found that higher levels of PA at admission predicted higher (rather than lower) discharge weights in patients with AN. In a similar study, Lehmann et al. (2018) found that overall PA and discharge weight were not associated; however, an estimate of “step counts” was linked to lower weight increases. Gianini et al. (2016) found that the “time spent on feet” at discharge predicted lower follow‐up weight, but earlier PA estimates had no effect on discharge weight. Finally, El Ghoch et al. (2013) found higher PA levels to predict dropout from AN treatment, potentially resulting in lower weight gain; however, this association was not directly explored. Thus, although there are some indications regarding the role of objective PA for early weight gain, the diverging findings and diversity of indices make it difficult to draw firm conclusions.

The role of subjective PA urges remains largely unexplored. The absence of investigations likely stems from a lack of instruments for the assessment of activity urges, which is why we recently developed and introduced the 21‐item State Urge to be Physically Active‐Questionnaire (SUPA‐Q; Amin et al. 2023). The SUPA‐Q captures activity urges in terms of an affectively charged momentary state to engage in (any) PA, characterized by psychological burden and further cognitive, emotional, and behavioral aspects on a 5‐point scale from 1 (not at all, 0%) to 5 (a great deal, 100%). Its design was inspired by patients with AN reporting acute “urges” to engage in PA (Casper et al. 2020; Graap, Erim, and Paslakis 2018), phenomenologically distinct from more specific, “trait‐like” addictions or compulsions for exercising found in patients with AN (Di Lodovico et al. 2021). An initial investigation among patients with eating disorders confirmed the SUPA‐Q's convergent validity with eating psychopathology and exercise dependence, its divergent validity from general impulsivity, and its state‐like properties, that is, its sensitivity to change. These findings verified the utility of the SUPA‐Q to assess momentary activity urges in patients with AN, adding to the diagnostic repertoire of a still scarce literature of activity urges measures (for nonclinical populations, see Stults‐Kolehmainen et al. 2021).

Extending upon our previous work, the present study investigated whether state activity urges, measured using the SUPA‐Q, could predict early within‐person weight gain during inpatient treatment for AN. We based our investigation on the clinical records of patients with AN included in the original validation study (Amin et al. 2023), for whom we extracted the routine weight data collected as part of inpatient treatment. Similar to previous studies (Chatelet et al. 2020; Halbeisen et al. 2024; Kolar et al. 2022), we defined early weight gain as the time‐dependent linear increase in body mass index (BMI) within the first 2 weeks of inpatient treatment, corresponding to the clinic's protocol which required patients to neither leave the clinic grounds, nor engage in exercise, or skip meals during the first 2 weeks. We hypothesized that more pronounced activity urges at treatment onset, indexed by a higher SUPA‐Q score, would predict lower within‐person early weight gain. Moreover, we explored which specific SUPA‐Q cutoff score at treatment onset could predict the recommended weight gain of 500 g per week, and whether the predicted changes in early weight gain could link state activity urges at admission to the patients' discharge weights.

## 2 Method

### 2.1 Sample

We included the clinical records of *N* = 53 adults with binge‐eating/purging AN (*n* = 22), restricting AN (*n* = 21), or atypical AN (*n* = 10; total sample: 51 women, two men; *M*_age_ = 25.15, age range: 18–64 years; *M*_BMI_ = 15.67, BMI range: 12.78–17.84 kg/m^2^), diagnosed by experienced clinicians according to ICD‐10 criteria, who had participated in our original validation study (Amin et al. 2023). Similar to previous studies (Kolar et al. 2022), the analysis only included patients with AN treated for at least 2 weeks who had a BMI < 18.5 kg/m^2^ at admission, for whom weight restoration was a primary treatment target (note that atypical AN based on ICD‐10 may include cases with underweight that do not fulfill other AN criteria). The original data collection was registered under https://aspredicted.org/YSZ_P53. The Ethics Committee of the Ruhr‐University Bochum's Medical Faculty at Campus East‐Westphalia approved the present analysis (AZ 2021‐799_2, February 9, 2024). All participants provided informed written consent during the original data collection.

### 2.2 Measures and Procedure

Patients were recruited from a specialty eating disorder facility in Germany, between November 2021 and October 2022, to receive a multimodal disordered eating treatment based on psychodynamic and cognitive‐behavioral approaches. They were recruited during the first 2 weeks of treatment, during which patients were required to neither leave the clinic grounds, nor to engage in exercise, or skip meals. Within this “protective phase”, patients are made aware and agree to be discharged should they be unable to follow the clinic's rules. These rules are imposed to strengthen the patient's responsibility and self‐efficacy.

Patients completed the SUPA‐Q questionnaire (Amin et al. 2023), sociodemographic questions (age, sex [female, male, other], language fluency, and level of education), and additional measures. The 21 items of the SUPA‐Q on current activity urges (e.g., “I have the urge to be physically active right now”) were rated on a 5‐point Likert‐like scale from 1 (not at all, 0%) to 5 (a great deal, 100%), and can be aggregated into a total score reflecting a single latent factor (Amin et al. 2023). The additional measures included the Eating Disorder Examination‐Questionnaire (EDE‐Q; Hilbert, Hoek, and Schmidt 2017), which contains 22 attitudinal items that can be combined into a global score of cognitive and behavioral ED symptoms over the last 28 days (e.g., restraint eating and weight/shape concerns), rated on a 7‐point scale (from 0, never, to 6, every day). Patients also completed the single‐factor Commitment to Exercise Scale (CES; Zeeck et al. 2017), with eight items rated on a visual analog scale (horizontal line of 12.2 cm) with bipolar adjectives on each end that assessed obsessive‐compulsive aspects of PA, and the 21‐item Exercise Dependance Scale (EDS; Müller et al. 2013), combined into a total score, which assessed “trait‐like” addictions and compulsions for exercising over the past 3 months on a scale from 1 (never) to 6 (always). Finally, participants completed the 15‐item Barratt Impulsiveness Scale (BIS‐15; Meule, Vögele, and Kübler 2011), rating self‐descriptions of impulsivity traits on a 4‐point scale from 1 (never applies to me) to 4 (always applies to me), which can also be combined into a total score.

All patients were weighed daily during the first weeks of treatment (each morning, including on weekends) and at least weekly until being discharged, using a calibrated scale (KERN & SOHN GmbH, Balingen‐Frommern, Germany). We extracted the available weight (and height) data for each patient, starting at the SUPA‐Q assessment, to examine early weight gain as well as patient BMI at discharge.

### 2.3 Statistical Analysis

The questionnaires were aggregated according to their convention (for means and SDs, see Table 1), Cronbach's α = 0.95 (SUPA‐Q), 0.95 (EDE‐Q), 0.94 (CES), 0.96 (EDS), and 0.66 (BIS). Missing responses were less than 1%, and thus, all scores could be computed. Within‐person weight gain (ICC = 0.42) served as our primary dependent variable, which we calculated using a random‐effects model using all available weight data (Halbeisen et al. 2024; Kolar et al. 2022). Specifically, we predicted patient BMI values for each weight measurement (*n* = 322 in total) obtained within the first 14 days (coded 0 to 13), starting at the SUPA‐Q assessment, with by‐participant random intercepts and random weight gain slopes. The (random) intercept represented the (predicted) admission BMI, and the (random) slope represented the average daily BMI change for each patient, with positive values denoting weight gain. We extracted the intercept and slope estimates for each patient to serve as dependent variables in further analyses (see below). For further exploration, we also calculated the root‐mean‐squared errors (RMSEs) per patient, that is, the standard deviation of the regression's residuals. The RMSEs represent the variation of BMI values along the regression slope and are an index of weight variability, which previous studies occasionally linked to reduced weight restoration (Chen, Singh, and Lowe 2021; Kolar et al. 2022, but see Halbeisen et al. 2024). However, because our sample's RMSE values were unrelated to the SUPA‐Q or other scores, and not predictive of discharge weights, these results are not reported in the text (see Table 2).

**TABLE 1:** Patient sociodemographic and descriptive statistics (mean with SD or n).

Parameter	Mean (SD, range) or *n* (%)
Age (years)	25.15 (9.74; 18–24)
BMI (admission)	15.67 (1.33; 12.78–17.84)
Early BMI gain (14 days)	0.03 (0.40; −0.67 to 1.15)
Treatment duration (days)	56.25 (15.79; 21–105)
Sex	
Male	2 (3.8%)
Female	51 (96.2%)
Other	0 (0%)
German language	
First language	51 (96.2%)
Fluent	2 (3.8%)
Education	
Less than 12 years	14 (26.4%)
12 years or more	39 (73.6%)
AN subtype	
Binge‐eating/purging	22 (41.5%)
Restricting	21 (39.6%)
Atypical	10 (18.9%)
Questionnaires mean scores	
SUPA‐Q	3.10 (1.02; 1.00–4.62)
CES	7.81 (3.16; 0.00–12.13)
EDS	3.51 (1.38; 1.00–5.93)
BIS‐15	1.88 (0.32; 1.20–2.93)
EDE‐Q global	4.10 (1.32; 0.86–5.82)
Mixed‐effects model estimates of early BMI gain	
Admission BMI (by‐participant random intercepts)	15.66 (1.36; 12.68–17.98)
Daily BMI gain (by‐participant random slopes)	0.002 (0.02; −0.05 to 0.07)
Within‐patient BMI variability (RMSE)	0.081 (0.05; 0.001–0.349)

Abbreviations: AN = anorexia nervosa, atypical = patients that do not fulfill the full range of AN criteria, binge‐eating/purging = AN subtype with binge‐eating or purging episodes, BIS‐15 = Barratt Impulsiveness Scale, BMI = body mass index in kg/m^2^, CES = Commitment to Exercise Scale, EDE‐Q = Eating Disorder Examination‐Questionnaire, EDS = Exercise Dependence Scale, restricting = AN subtype without binge‐eating and purging, RMSE = root‐mean‐squared error of individual‐level regressions, SUPA‐Q = State Urge to be Physically Active‐Questionnaire.

**TABLE 2:** Results of multiple linear regressions of daily BMI gain (top panel) and variability (lower panel) on SUPA‐Q scores, controlling for admission BMI, patient age (mean‐centered), and AN subtype (active binge/purge vs. restricting vs. atypical, dummy‐coded, with restricting as reference).

Variable	*B*	SE	Beta (*β*)	*t*	*p*
Daily BMI gain (slopes)					
Constant	0.064	0.036		1.774	0.083
SUPA‐Q	−0.009	0.003	−0.408	−2.924	0.005
Admission BMI	−0.002	0.002	−0.104	−0.786	0.436
Age (centered)	0.000	0.000	−0.078	−0.585	0.561
AN binge/purge	−0.012	0.007	−0.254	−1.805	0.078
AN atypical	−0.003	0.009	−0.045	−0.290	0.773
BMI variability (RMSE)					
Constant	0.211	0.099		2.123	0.039
SUPA‐Q	−0.005	0.009	−0.101	−0.606	0.547
Admission BMI	−0.007	0.006	−0.179	−1.184	0.242
Age (centered)	0.000	0.001	−0.064	−0.416	0.680
AN binge/purge	0.004	0.018	0.035	0.215	0.831
AN atypical	−0.001	0.026	−0.010	−0.057	0.955

*Note*: Overall model tests: *F*(5, 47) = 3.49, *p* = 0.009, adj. *R* ^2^ = 0.19 (top panel), and *F*(5, 47) = 0.36, *p* = 0.87, adj. *R* ^2^ = −0.06 (lower panel).

We conducted three sets of analyses using the slopes of average daily BMI change. First, we used multiple linear regression to investigate whether SUPA‐Q scores predict daily BMI gain, controlling for admission BMI estimate (i.e., the random intercept), patient age (mean‐centered), and AN subtype (binge/purge vs. restricting vs. atypical, dummy‐coded) as covariates. We then separately entered global symptom severity scores (EDE‐Q), trait‐like compulsive and addicted exercise behaviors (CES, EDS), and impulsiveness (BIS) into the model (all mean‐centered) and tested for improvements in model fit (R^2^ change) to evaluate alternative explanations for the SUPA‐Q's predictive effect (i.e., the SUPA‐Qs incremental validity for predicting early BMI gain). Because these tests were run separately to evaluate independent hypotheses for each construct assessed by the questionnaires, we did not make any corrections to α (Rubin 2021). Second, we explored which specific SUPA‐Q cutoff score could predict the recommended weight gain of 500 g per week (calculated using daily BMI slopes and patient height) in a receiver‐operating‐characteristic (ROC) analysis. Finally, we explored whether the predicted changes in early BMI gain would link state activity urges at admission to the patients' discharge BMI in a mediation analysis, controlling for admission BMI estimate, admission symptom severity (EDE‐Q, mean‐centered), age (mean‐centered), AN subtype, and treatment duration (mean‐centered) as covariates.

The significance level for all analyses was set at *p* ≤ 0.05. Variable values are reported as means (M) and standard deviations (SDs). The data were aggregated and analyzed with IBM SPSS 28 (IBM Corp. 2021). We used PROCESS v4.2 (Hayes 2022) with bootstrapped (10,000 samples) bias‐corrected 95% confidence interval (CI) to explore mediation patterns. Random‐effects models for daily weight gain slopes were computed using package lme4 1.1.35.1 (Bates et al. 2015) in R 4.3.2 (R Core Team 2022).

## 3 Results

**FIGURE 1:**
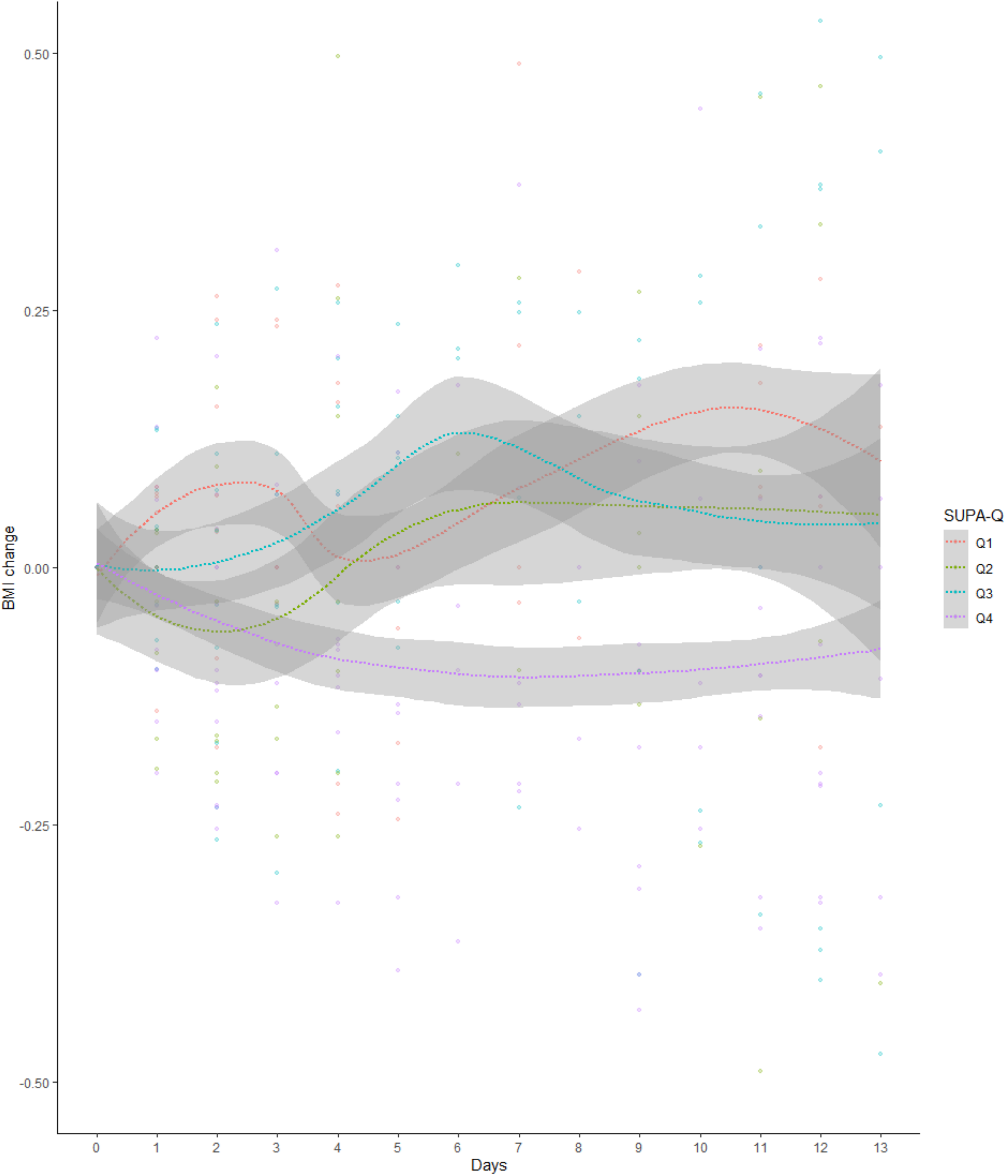
BMI change (differences from admission BMI per patient, see Dots) smoothed regressions lines by SUPA‐Q quartiles (splits at 2.11, 3.09, and 3.81). Bands show the standard errors.

Table 1 shows the patients' demographic data, questionnaire scores, and estimates extracted from the random‐effects model. The initial regression model explained a significant amount of weight gain variance, *adj. R* ^2^ = 0.19, *F*(5, 47) = 3.49, *p* = 0.009. For every 1‐point increase in SUPA‐Q mean score, the average daily BMI gain was reduced by *b* = −0.01 kg/m^2^, SE = 0.003, *t*(47) = −2.92, *p* = 0.005, the equivalent of 184 g per week in the present sample (see Figure 1). The effects of age, admission BMI, and AN subtype were not significant, *p*s > 0.07. Including the scores for global symptom severity (EDE‐Q), compulsions for exercising (CES), exercise dependence (EDS), and impulsiveness (BIS) into the model could not explain any additional variance beyond the SUPA‐Q in weight gain slopes, *R* ^2^ *change* = 0.001, 0.004, 0.02, and 0.005, respectively, all *p*s > 0.23.

**FIGURE 2:**
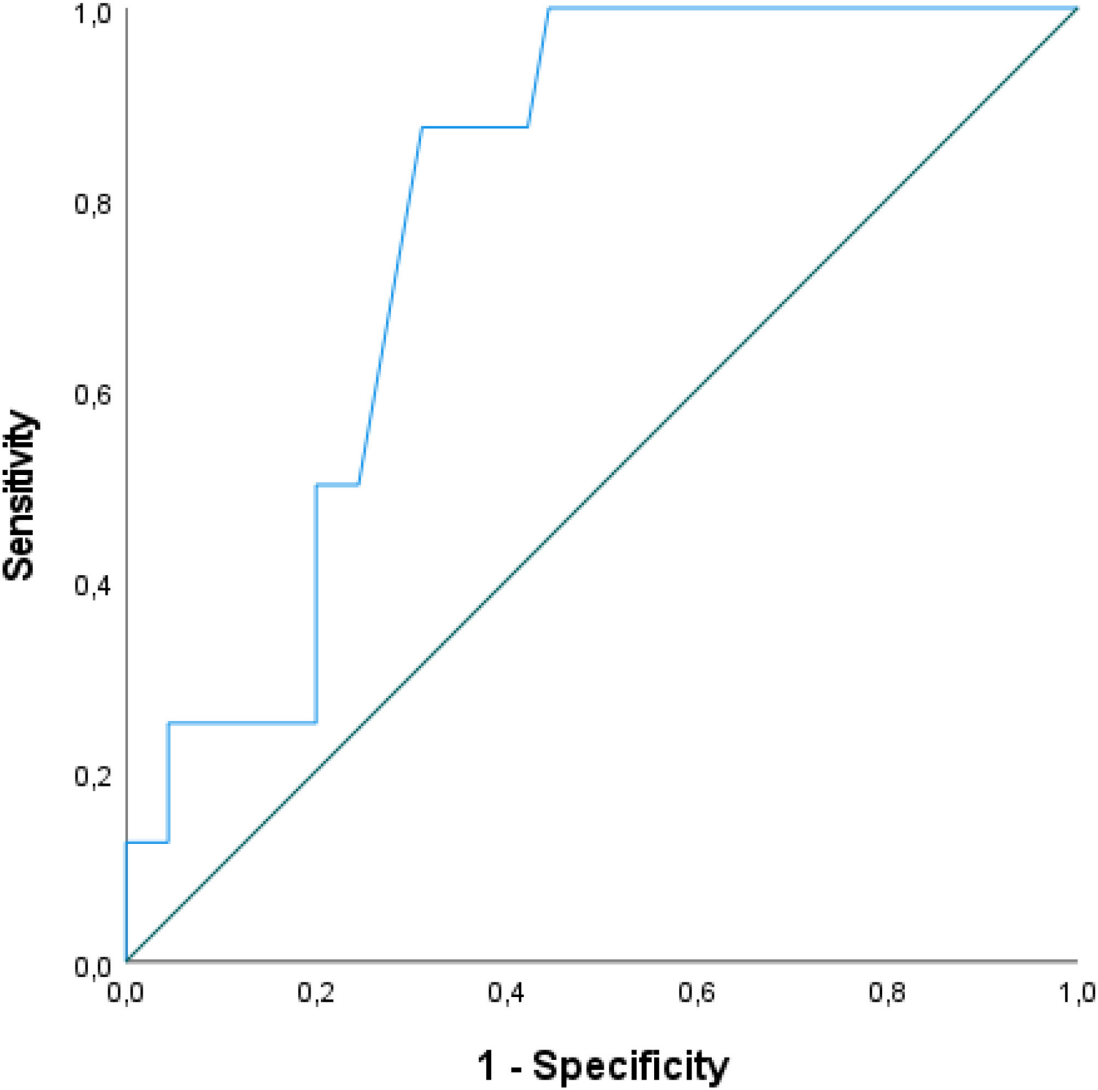
Receiver‐operating‐characteristic (ROC) curve for discriminating between patients with AN reaching vs. not reaching the required 500 g weight gain per week based on the SUPA‐Q mean scores.

The estimated average weight gain per week across patients was 34.2 g (SD = 481.81; range: −974 to 1565 g) and normally distributed, *W* = 0.98, *p* = 0.61. Eight patients gained the recommended 500 g per week or more, and 45 remained below this threshold (of those, 19 gained weight and 26 lost weight). The ROC analysis (see Figure 2) with SUPA‐Q scores as the predictor of weight gain success showed an area under the curve (AUC) of 0.79, 95% CI [0.66; 0.91], suggesting fair diagnostic accuracy (Nahm 2022). The Youden Index determined a SUPA‐Q score of 2.76 as the optimal cutoff for weight gain success, with a sensitivity of 0.88 and a specificity of 0.69, to distinguish between positive (successful) cases (SUPA‐Q score less than or equal to 2.76) and negative (unsuccessful) cases (SUPA‐Q score higher than 2.76).

**FIGURE 3:**
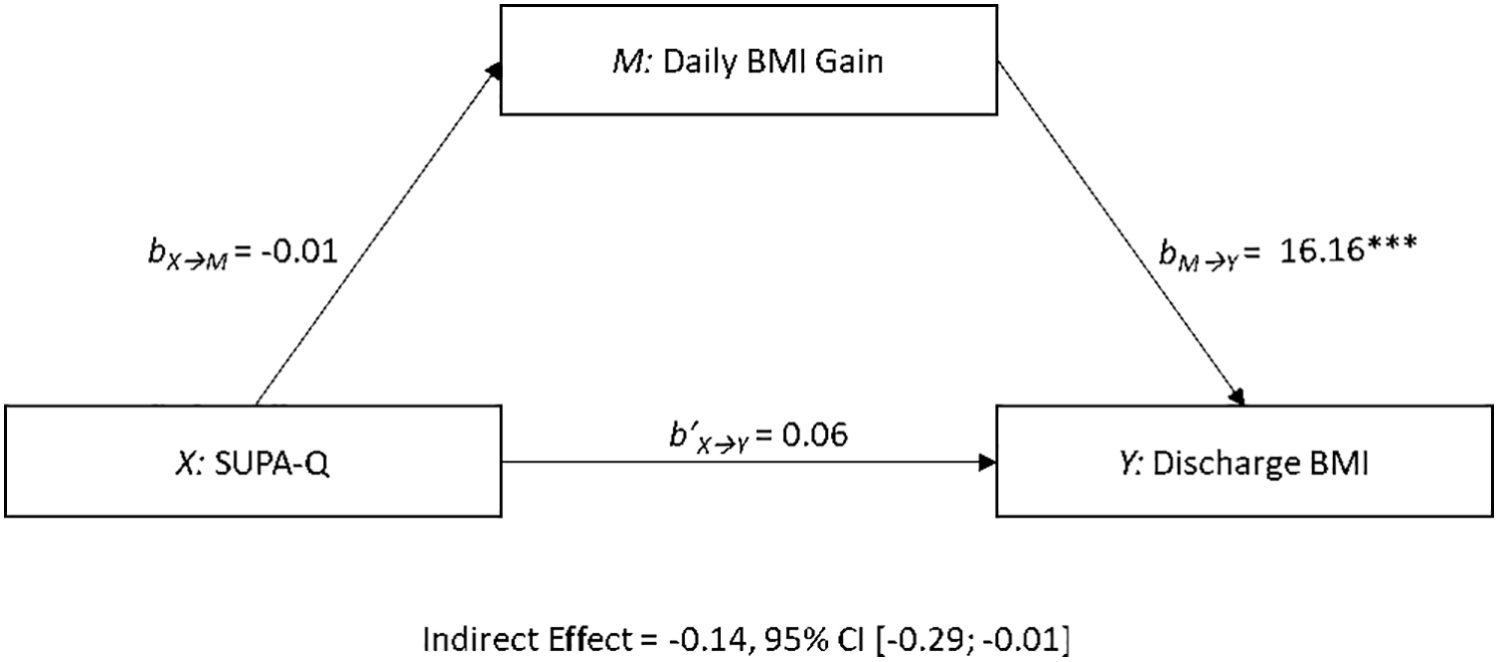
The mediation model with state activity urges at admission as predictor X, daily BMI gain during the first 2 weeks of treatment as mediator M, and discharge BMI as criterion Y (with admission BMI, EDE‐Q, patient age, AN subtype, and treatment duration as covariates).

Finally, a mediation analysis (see Figure 3) explored whether the predicted changes in early BMI gain (mediator *M*) would link state activity urges at admission (predictor *X*) to the patients' discharge BMI (criterion *Y*), controlling for admission BMI estimate, admission symptom severity (EDE‐Q, mean‐centered), patient age (mean‐centered), AN subtype, and treatment duration (mean‐centered) as covariates. The bootstrapped analysis revealed a significant indirect effect of SUPA‐Q scores on discharge BMI via changes in weight gain slopes, −0.14, 95% CI [−0.29; −0.01], suggesting that a 1‐point increase in SUPA‐Q mean scores was associated with an average reduction in discharge BMI by 0.14 points (the estimated equivalent of 406 g in the present sample).

## 4 Discussion

Activity urges have been suspected to delay early weight gain during AN treatment (Graap, Erim, and Paslakis 2018; Marzola et al. 2013; Paslakis et al. 2017). Using the newly developed SUPA‐Q, this study investigated the prognostic role of state activity urges for predicting within‐person weight changes in adults with AN from an inpatient unit during the first 2 weeks of treatment. We found that more pronounced activity urges were associated with lower weight gain, not further explained by exercising compulsions, addiction‐like exercise behaviors, general psychopathology, or impulsiveness, equivalent to a difference of 184 g per week for every 1‐point increase in SUPA‐Q scores (i.e., a large effect by convention, considering the SD of the SUPA‐Q is 1.02; differences of 38–94 g would be expected for small to medium‐sized changes in SUPA‐Q scores). A mean cutoff score below or above 2.76 (on a scale from 1 to 5) distinguished between patients reaching vs. not reaching the recommended 500 g weight gain per week with fair diagnostic accuracy (Nahm 2022). We further found that the predicted changes in weight gain trajectories during early treatment linked more pronounced activity urges at treatment onset to lowered discharge weights, controlling for treatment duration, by 0.14 BMI points (equivalent to 406 g in our sample) for every 1‐point increase in SUPA‐Q scores (or 83–207 g for small to medium‐sized changes in SUPA‐Q scores). Thus, the present findings support the idea of a prognostic role of acute activity urges for weight gain outcomes.

One plausible interpretation of these findings is that state active urges motivate objective PA levels in terms of both exercising and nonexercising behaviors (e.g., fidgeting), leading to increased energy expenditure and thus lower weight gain (Chung et al. 2018; Elbelt et al. 2013; Haas et al. 2018; Jakicic 2009; Westerterp 2013). Indeed, objective activity based on (proprietary) actigraphy measures has been linked to reduced weight gain in patients with AN (El Ghoch et al. 2013; Lehmann et al. 2018), for which activity urges could serve as a low‐cost, open‐source proxy. Activity urges could also result from neuroendocrine changes due to underfeeding (Casper 2006) and thus mark the overall severity or duration of AN pathology. Thus, further research on the mediating mechanism and causal role of activity urges in predicting weight gain is needed.

The evidence for a prognostic role suggests activity urges to be a potential intervention target to facilitate weight restoration. Although AN treatment may often include guided exercising sessions at later stages of recovery (Hallward, Di Marino, and Duncan 2021), the subjective burden related to the initially prescribed inactivity (Brunet, Del Duchetto, and Wurz 2021) remains largely unaddressed and calls for novel approaches. For example, Paslakis et al. (2017) explored whether simulated movement within a virtual reality environment, through the illusion of actual body movement, could alleviate activity urges in patients with AN (and Bulimia Nervosa). The authors found a 14% reduction in acute activity urge scores from baseline after a 30‐min session, pointing toward a potential utility of simulated activities in AN treatment. However, further studies are needed to explore the causal mechanisms of activity urges in eating disorders, and interventions need to account for such mechanisms to avoid being ineffectual or even counterproductive.

### 4.1 Strengths and Limitations

The present study used a clinical sample of various diagnosed AN subtypes to identify factors beneficial or detrimental to early weight gain based on a novel measure of state activity urges. We evaluated the predictive validity of the SUPA‐Q against established weight gain criteria and provided effect size estimates and cutoff scores that allow for an easier application of the results in clinical practice. Although we caution against premature conclusions, the study highlights avenues for future research that may ultimately help to improve weight gain in patients with AN.

Of course, we must also note some limitations. We did not measure objective PA levels or other variables related to the assumed underlying mechanism of the effect of activity urges. We also recruited individuals with AN in a highly controlled inpatient setting, and our findings thus cannot speak to the overall importance of activity urges for weight restoration in other settings. It is conceivable that the perceived restrictions in the inpatient setting could have increased the subjective burden of activity urges, and thus aggravated their effects. Relatedly, our sample was rather homogeneous concerning sex, education, and language fluency, raising the question of whether the obtained results and specific cutoff values can be replicated in other populations. We must also note that we did not assess patients' gender identity, sexual orientation, income, or other potentially relevant comorbidities, which could be linked to the severity of AN psychopathology as well as weight gain.

Our analysis was further limited to short‐term weight changes and did not include any long‐term follow‐up. Although early weight gain has been identified as an important predictor of positive clinical outcomes, weight restoration in patients with AN often remains only temporary, with a majority of studies reporting relapse rates greater than 25% and up to 52% within the first year after successful treatment (Khalsa et al. 2017). Thus, claims to the prognostic role of activity urges require further long‐term validation. Ideally, this would also include repeated assessments of activity urges throughout AN treatment. We designed the SUPA‐Q to measure current motivational states, and the previous validation study confirmed the scale's sensitivity to change (Amin et al. 2023). However, given the retrospective nature of our study, we could only rely on a single measurement at admission for the selected sample. It is possible that activity urges in patients with more pronounced SUPA‐Q scores were more likely to change during treatment and that changes in activity urges (rather than their initial level) exert a prognostic effect.

Finally, we must address some methodological limitations. The present analysis used parts of the original SUPA‐Q validation sample (Amin et al. 2023); thus, the psychometric properties of the SUPA‐Q should be further explored in other samples. Given its retrospective nature, we also did not conduct an a priori power analysis. An a posteriori power analysis showed the sample had 0.93 chance of finding the observed effect of the overall regression model (adj. *R* ^2^ = 0.19). However, the power for a medium‐sized effect would have been lower (1 – *β* = 0.43), suggesting a need for further replication.

## 5 Conclusions

The present findings support the idea of acute activity urges as a prognostic factor for weight restoration in patients with AN. Measured activity urges predicted lower weight gain during the initial weeks of treatment, which further linked activity urges to the patients' discharge weights. Future studies need to explore the underlying mechanisms of the observed effect and evaluate the utility of addressing activity urges for patients' treatment outcomes.

## Author Contributions

**Georg Halbeisen:** conceptualization, data curation, formal analysis, visualization, writing – original draft. **Lina Amin:** data curation, validation, writing – original draft. **Karsten Braks:** data curation, writing – review and editing. **Thomas J. Huber:** resources, writing – review and editing. **Georgios Paslakis:** project administration, supervision, writing – review and editing.

## Conflicts of Interest

The authors declare no conflicts of interest.

## Data Availability

The data that support the findings of this study are available from the corresponding author upon reasonable request.
